# Staphylococci: What Has Changed in the Antibiotic Resistance Profile in the Last Decade—Analysis of Strains Isolated from Hospitalised Patients

**DOI:** 10.3390/pathogens14121289

**Published:** 2025-12-15

**Authors:** Alessio Facciolà, Antonio Laganà, Maria Eufemia Gioffrè, Alessandro Morabito, Domenico Chiera, Marco Ferlazzo, Pasqualina Laganà

**Affiliations:** 1 Department of Biomedical Sciences Dental Sciences and Morpho-functional Imaging, University of Messina, Via C. Valeria, 98125 Messina, Italy; afacciola@unime.it (A.F.); antonio.lagana1@studenti.unime.it (A.L.); alessandro.morabito1@studenti.unime.it (A.M.); 2 Multispecialty Clinical Institute, Trauma Orthopedic Care, Via Ducezio 1, 98124 Messina, Italy; emigioffre@gmail.com (M.E.G.); d.chiera@cotmessina.it (D.C.); marco.ferlazzo@humanitascot.it (M.F.)

**Keywords:** *Staphylococcus* spp., antibiotic resistance, bacterial pathogenesis

## Abstract

Antibiotic resistance can be considered one of the major bacterial pathogenesis factors, and its increase is an important public health concern. Among resistant bacteria, staphylococci can express high levels of resistance, representing some of the most common human pathogens that are important causes of infection at any level. This paper aims to assess the burden of staphylococcal infections, in light of the growing phenomenon of antibiotic resistance, in a high-risk hospital setting. We carried out a cross-sectional study on data obtained from clinical specimens from an Italian healthcare facility particularly specialised in orthopaedics and traumatology. Specifically, we evaluated the trends in the *Staphylococcus* spp. detection rates and antibiotic resistance in the ten-year period of 2012–2024. The number of specimens increased over time but remarkably decreased during the COVID-19 pandemic. Despite this, the detection rate of *Staphylococcus* spp. remained quite constant, confirming the common circulation of these pathogens in such nosocomial environments. Antibiotic resistance concomitantly decreased, but a novel increase was found in recent years. Our findings highlight the importance of continuous monitoring of the antibiotic resistance rates of staphylococci due to the importance of these pathogens in human pathology and their ability to cause life-threatening antibiotic-resistant infections.

## 1. Introduction

Staphylococci are Gram-positive bacteria commonly commensal in human skin and other body parts and have with an important role in human pathology. They are classified into two groups according to the presence of the enzyme coagulase: *Staphylococcus aureus*, the main coagulase-positive species, and coagulase-negative staphylococci (CoNS), including many different species such as *S. epidermidis*, *S. capitis*, *S. haemolyticus*, and *S. lugdunensis*. *S. aureus* shows higher virulence than CoNS due to the production of numerous virulence factors, including exotoxins, exoenzymes, adhesins, and immune evasion factors [1,2].

Methicillin-resistant *S. aureus* (MRSA) and methicillin-resistant CoNS (MRCoNS) are important causes of both hospital- and community-acquired infections due to their mortality, morbidity, incurring high healthcare costs [3,4,5]. Staphylococci especially skin and soft tissue infections (SSTIs), but these bacteria can also reach deep organs and the bloodstream, causing systemic infections mainly of nosocomial origin, connected with the presence of medical devices [6,7,8]. Apart from SSTIs, *S. aureus* is considered one of the most important pathogens in osteo-articular issues, such as osteomyelitis, prosthetic joint infections, and joint arthritis [9,10,11,12], and for this reason, facilities specialised in orthopaedic and traumatology surgery are settings that place patients at high risk of developing these kinds of infections [13]. Moreover, it is important to underline that, unlike other *Staphylococcus* species, *S. aureus* can be highly pathogenic even in the absence of predisposing host conditions, such as general or local immunosuppression or immunodeficiency [14].

The methicillin resistance of staphylococci is a serious concern in the treatment of these infections. For both MRSA and MRCoNS infections, vancomycin is currently the first-line drug for treatment. However, in recent years, an increased resistance to vancomycin and other antibiotics, such as tetracyclines, aminoglycosides, and lincosamides, has been reported worldwide [15,16,17]. Consequently, for the management of severe infections caused by multi-drug-resistant staphylococci, second-line molecules such as linezolid and tigecycline were introduced [18].

Linezolid is an antibiotic belonging to the oxazolidinones, which was approved in 2000 for the treatment of infections caused by MRSA, MRCoNS, and vancomycin-resistant enterococci (VRE).

Tigecycline is a new broad-spectrum antimicrobial agent belonging to the glycylcyclines active against Gram-positive and Gram-negative bacteria. This antibiotic was approved by the U.S. Food and Drug Administration (FDA) in 2005–2009 for the treatment of skin infections, intra-abdominal infections, and community-acquired bacterial pneumonia [19,20]. Tigecycline is an important alternative choice for the treatment of complicated MRSA and VRE infections.

For all these reasons, it appears particularly crucial to continuously monitor the spread of these dangerous resistant strains in different health settings to rapidly identify even small potential clinical outbreaks and implement specific preventive tools in order to avoid their spread to the community.

The aim of this study was to evaluate the burden of staphylococcal infections in a particularly critical setting, a hospital area specialised in orthopaedic and traumatological surgery, particularly investigating the trend in detecting antibiotic-resistant strains from human clinical samples.

## 2. Materials and Methods

### 2.1. Setting and Samples

We conducted a retrospective laboratory-based observational study on clinical specimens from a healthcare setting in the southern Italian city of Messina, Sicily. This healthcare facility is a multidisciplinary clinical institute but particularly specialised in orthopaedics and traumatology, including 9 different medical, surgical and rehabilitative wards with 91 hospital beds. The hospital is a private facility but belongs to the National Health System network and is accredited with the Joint Commission International system. The patients who seek treatment at the facility are mainly individuals who require orthopaedic and traumatological procedures, given the facility’s specialisation. These patients include both older adult patients hospitalised for fractures of the femur or other bones and young adults who require surgery, mainly following traumatic events. Since 2011, this important healthcare facility has been covered and supported by our microbiology laboratory, for analysing human and environmental microbiological. The biological samples sent to our laboratory are mainly surgical wound swabs, urine, vascular or diabetic ulcer swabs, prosthetic material, synovial fluid, etc. Specifically, we investigated the detection rates of *Staphylococcus* spp. strains and their antibiotic susceptibility from biological samples sent in the period of 2012–2024.

### 2.2. Microbiological Analysis

From a general point of view and in everyday laboratory practice, the clinical samples arriving to our laboratory are tested through routine microbiological assays to determine the presence of Gram-negative and Gram-positive bacteria and their antibiotic susceptibility. Specifically, the samples are grown on classic culture media such as 5% Columbia blood agar, cystine–lactose–electrolyte-deficient (CLED) agar, Mannitol–Salt Agar (MSA), Hektoen enteric agar, and SS agar (ThermoFisher, Waltham, MA, USA). After incubation at 37 °C for 24 h, suspected colonies are further analysed and finally identified by a Vitek-2 Compact system (bioMérieux, Marcy l’Etoile, France), according to the manufacturer’s instructions. The same system is used to obtain antibiotic susceptibility/resistance profiles. However, for research purposes, in order to increase the range of antibiotics used, in addition to the automated method, the manual Kirby–Bauer method is used. To this aim, the bacterial material is suspended in sterile water in order to obtain a 0.5 McFarland turbidity standard solution, corresponding to 1.5 × 10^8^ CFU ml^−1^, and the suspension is inoculated on plates with Mueller–Hinton agar (ThermoFisher, Waltham, MA, USA). Then, antibacterial discs are used to determine the resistance patterns of the isolates against different antibiotics (listed in Table S1), grouped according to their activity (cell wall inhibitors, disruptive membrane antibiotics, nucleic acid inhibitors, and protein synthesis inhibitors) (ThermoFisher, Waltham, MA, USA) [21].. Each bacterial species was classified as resistant (R), intermediately resistant (I), or sensitive (S) according to the breakpoints established by the European Committee on Antimicrobial Susceptibility Testing (EUCAST) [22] and the Clinical & Laboratory Standards Institute (CLSI) [23].

### 2.3. Statistical Analysis

All the obtained data were collected and analysed with Prism 4.0 software (GraphPad, San Diego, CA, USA). Descriptive statistics were used to calculate mean values, standard deviations, and percentages. Fisher’s exact test was used to compare percentage values and establish significant differences between variables. Significance was set at the *p* < 0.05 level.

## 3. Results

**Figure 1:**
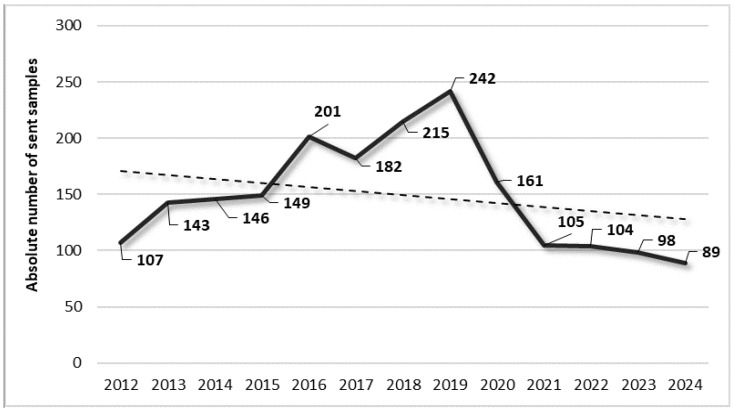
Trend in the absolute number of samples sent to the laboratory during the period of 2012–2024.

In the considered period, 1942 clinical specimens were sent to the laboratory. The trends and details are shown in Figure 1.

**Figure 2:**
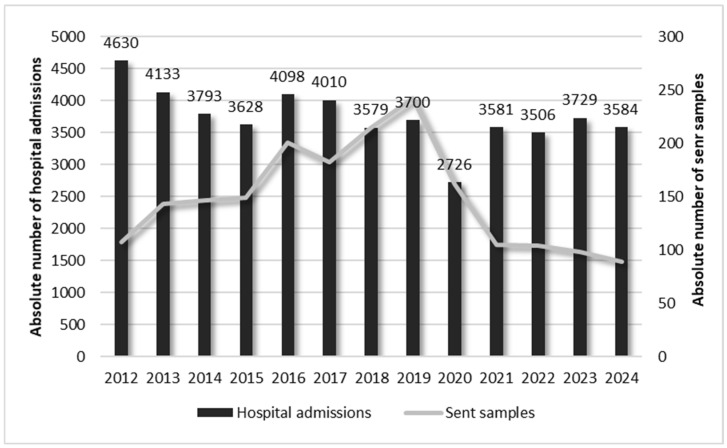
Comparison between hospital admissions and number of samples sent to the laboratory in the period of 2012–2024.

The figure shows that after six years of an overall constant and progressive rise from 2012 to 2019, there was a remarkable reduction during the two-year pandemic period of 2020–2021, decreasing by −33.5% and −56.6% compared to 2019, respectively. In order to understand if the first increase was absolute and did not correlate with fluctuations in the hospital admissions, we evaluated the number of the latter during the considered period of time. This analysis confirmed that the increase was absolute, as the number of hospital admissions in the same period slightly decreased. Indeed, against a percentage decrease of −20.1% observed for hospital admissions, there was a parallel increase of +65.7% in the number of sent samples. The situation was completely different during the two-year pandemic period of 2020–2021 when, due to a reduction of −26.3% in the hospital admissions from 2020 to 2019 (because the clinic was converted into a centre specifically dedicated to respiratory rehabilitation of individuals affected by COVID-19), there was a parallel decrease of −33.5% in the sent samples. However, from 2020 to 2024, despite a partial increase in hospital admissions of +31.5%, there was a reduction of −44.7% in the number of samples sent to the laboratory (Figure 2).

Of all the sent samples, 196 (10.1%) tested positive for *Staphylococcus* spp., of which 121 (61.7%) and 75 (38.3%) were from men and women, respectively. The average age of the patients was 66 ± 16 years (min. 2–max. 98). Of the total patients, 111 (70.2%) suffered from co-morbidities and/or risk conditions for infections, of which 57.1% was diabetes, 14.4% cardiovascular diseases, 14.3% liver diseases, 7.1% chronic obstructive pulmonary disease (COPD), and 7.1% bone fractures. Table 1 shows the number and percentages of the *Staphylococcus* spp. detection rates per year.

The table shows that one-tenth of the sent samples tested positive for *Staphylococcus* spp., with a fluctuating trend. Actually, after an initial decrease of −35.7% in the first four years (2012–2015) of analysis, we detected a remarkable and constant increase in the next four years (2016–2019). In the pandemic period (2020–2022), a new, important decrease of −94.4% was detected, followed by a slight increase in the last two years.

**Figure 3:**
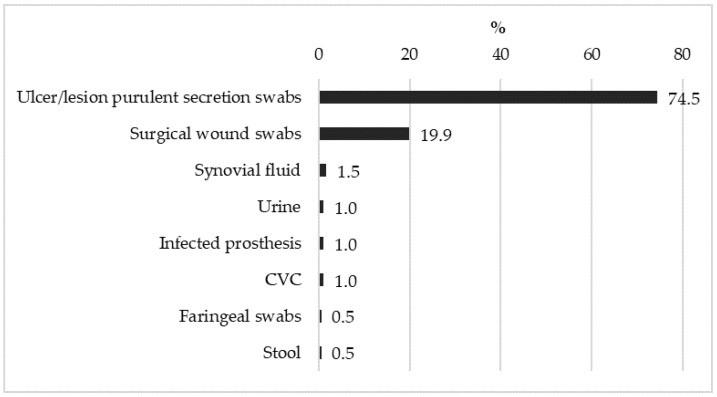
Percentages of sample types where bacteria were detected.

The range of samples in which staphylococci were detected was particularly wide, for the most part from swabs of different origins. The results are summarised in Figure 3.

The figure shows that the swabs were by far the most frequent type of sample, representing the 94.9% of the total. They were mainly swabs of purulent secretions from ulcers and lesions, particularly of vascular, diabetic, and bedsore origin. A particular mention has to be made for swabs from surgical wounds, which showed surgical site infections (SSIs), which represented almost 20% of the samples. These swabs were taken from patients who had undergone surgery, mainly of orthopaedic and trauma natures, given the specialised nature of the clinic. In this group of patients, there were no significant age or gender differences compared to patients affected by community infections. Specifically, the mean age of the SSIs group was 63.2 ± 14.9 (min. 23.0, max. 92.1) compared to 68.1 ± 14.2 (min. 11.0, max. 98.2) years in the community infection group. About gender, 64.1% of the SSIs group were men, compared to 61.1% men in the community infections group.

**Figure 4:**
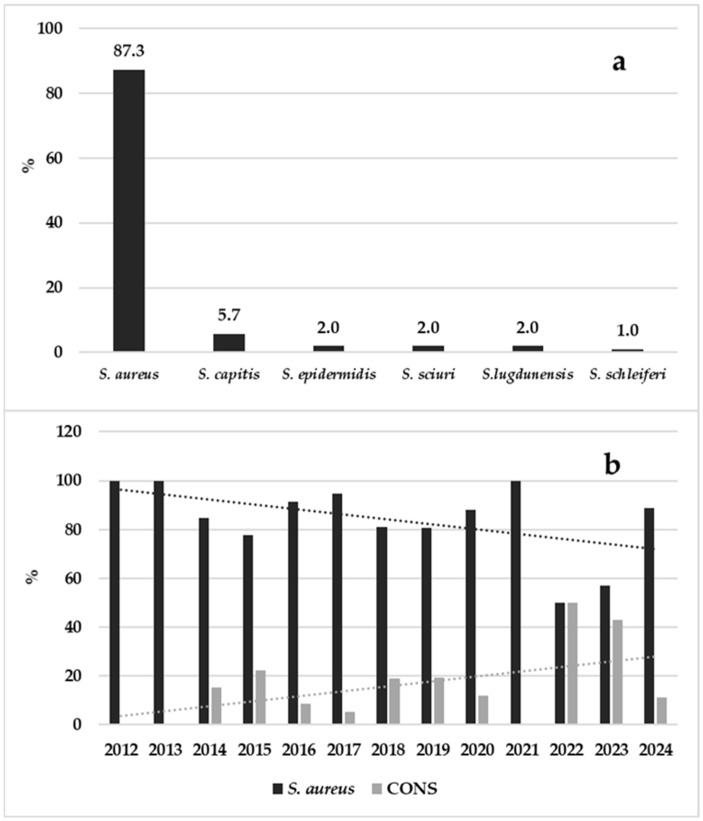
Detail of detected staphylococcal species (**a**) and difference in the detection rates of *S. aureus* and CoNS (**b**).

About microbial aetiology, *S. aureus* was by far the most-detected species (87.3%), and CoNS represented just over one-tenth of the infections (12.7%). Overall, a fairly constant trend was observed in the detection of the two types of microorganisms, with the exception being the two-year period of 2022–2023, when there was a decrease in the detection of *S. aureus* and a parallel increase in CoNS detection. The details of the detected species and the differences in the detection rates are shown in Figure 4.

Despite the lack of age or gender differences found between SSIs and community infections, a significant difference was found in aetiology. Specifically, *S. aureus* caused most (89.8%) of the community infections compared to causing 76.9% of SSIs (*p* = 0.0212). Of the 23.1% of CoNS causing SSIs, the species detected in decreasing order were *S. capitis* (32.1%), *S. schleiferi* (29.0%), *S. epidermidis* (18.1%), *S. sciuri* (10.1%), *S. lugdunensis* (9.1%), and *S. haemolyticus* (1.6%). Additionally, 50% of the patients with SSIs were undergoing antibiotic treatment compared to 28.8% of the patients with community infections (*p =* 0.0037).

**Figure 5:**
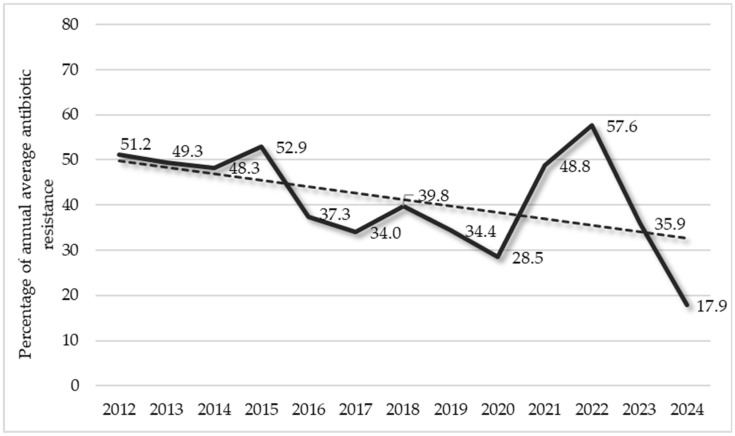
Trend in the overall antibiotic resistance to all tested antibiotics of the detected strains during the considered period.

In order to establish the level of risk posed to patients affected by staphylococcal infections in our study context, we evaluated the susceptibility/resistance patterns of the detected strains to the used antibiotics. Figure 5 shows the general trend in the mean annual average antibiotic resistance values of the detected strains during the considered period.

The figure was obtained by considering the average resistance values for each year for all antibiotics tested. In other words, the staphylococcal strains detected in 2012 were found to be resistant to 51.2% (just over half) of the antibiotics tested, as an average value, and this was the same for each year evaluated. Based on this parameter, the figure shows an overall decreasing trend in the average annual antibiotic resistance, with a remarkable decrease of −65.0% from 2012 to 2024. This decrease was constant until 2020 (Δ% = −44.3%). A remarkable increase (Δ% = +102.1%) was registered in the two-year period of 2021–2022 during the COVID-19 pandemic. In the last two considered years, the trend decreased again (Δ% = −68.9%).

**Figure 6:**
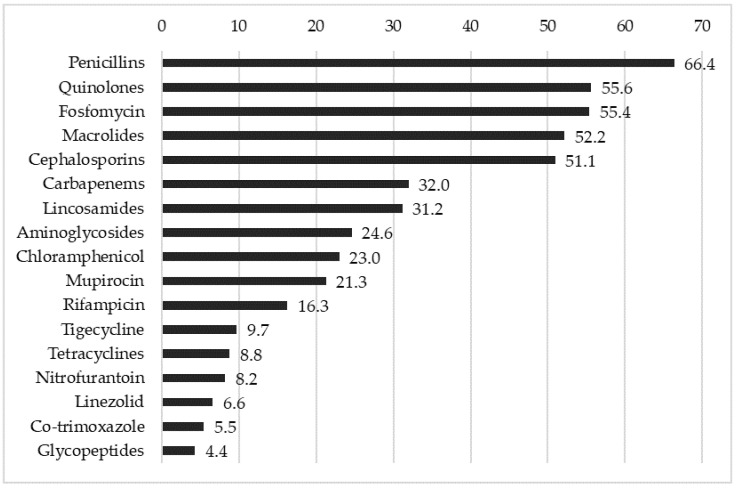
Resistance patterns of detected *Staphylococcus* spp. strains to different classes of tested antibiotics.

After assessing the overall resistance level of the detected strains, we stratified the results by evaluating the resistance to the individual classes of the antibiotics used (Figure 6).

On average, the resistance rate to all the tested classes of antibiotics was 27.8%. It is important to note that only 1.5% of the detected strains were sensitive to all antibiotics and showed no resistance. Furthermore, the vast majority (93.9%) of the detected strains were found to be multi-drug-resistant (MDR) according to the common definition that a bacterial strain is considered MDR when it is resistant to at least one antibiotic from three or more different antimicrobial categories. The range of resistance values was wide. The highest values were found for a large group of commonly used antibiotics such as penicillins, quinolones, and cephalosporins. We further divided these results, stratifying the data according to the resistance to the main subclasses, as shown in Table 2.

**Figure 7:**
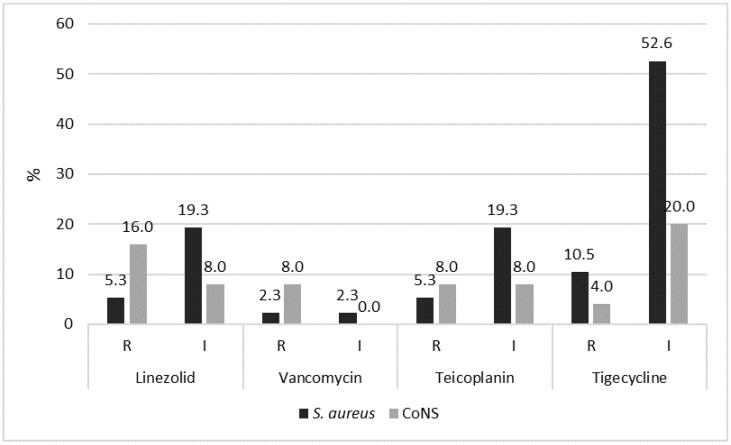
Resistance (R) and intermediate (I) patterns of the detected strains to the main first-line antibiotics used against MRSA and MRCoNS.

Among the penicillins, benzylpenicillin, penicillinase-resistant penicillins (methicillin and oxacillin), and aminopenicillins (ampicillin, amoxicillin, and amoxicillin–clavulanic acid) were those with the highest rate of resistance, while the resistance rates were lower for the newest carboxypenicillins (carbenicillin) and ureidopenicillins (mezlocillin and piperacillin). A special analysis was made for strains resistant to penicillinase-resistant penicillins, key molecules to consider in order to evaluate the level of hazard posed by this bacterial group. Specifically, 71.3% of *S. aureus* and 68.0% of CoNS were MRSA and MRCoNS, respectively. Finally, we extended our evaluation to the resistance patterns towards the first-line drugs used against infections caused by MRSA and MRCoNS: linezolid, teicoplanin vancomycin, and tigecycline, in the detected *S. aureus* and CoNS (Figure 7).

From the figure it is clear that CoNS showed higher levels of resistance to linezolid (*p* = 0.0192), vancomycin (no significant difference), and teicoplanin (no significant difference) than *S. aureus*. The opposite situation was found for tigecycline, resulting in *S. aureus* being more resistant than CoNS (no significant difference). For strains with intermediate behaviour, VISA (vancomycin-intermediate *S. aureus*) detection rates were very low (2.3%, no significant difference compared to CoNS), while a higher percentage (19.3%) was found for linezolid-intermediate and teicoplanin-intermediate *S. aureus* (*p* = 0.037). More than the half (52.6%) of *S. aureus* were classified as tigecycline-intermediate (*p* < 0.00001 compared to CoNS). These results were further divided according to the origin of the strains (SSIs and community infections). The results are shown in Table 3.

The table shows some remarkable differences between the strains detected from SSIs and those detected from community cases. We have to mention the significant difference in resistance to carbapenems and cephalosporins; strains detected from SSIs cases were more resistant to both of them. In general, SSIs strains were more resistant than community strains, except to quinolones, nitrofurantoin, and mupirocin, with no significant differences.

## 4. Discussion

Hospital settings specialised in orthopaedics and trauma surgery are particularly at risk of outbreaks of healthcare-associated infections (HAIs) caused by staphylococci, and for this reason it is essential to continuously monitor the presence and spread of these pathogens and the relative resistance/susceptibility patterns.

In this study, we evaluated the detection rates and the antibiotic susceptibility of *Staphylococcus* spp. strains isolated from human specimens collected from a large healthcare facility specialised in orthopaedics and traumatology. Our results highlight some critical issues that are important to remark and on, which require a detailed analysis. First of all, we found a gap in sending samples between the pre-COVID-19-pandemic and the post-pandemic periods. Indeed, a remarkable increase in the samples sent to our laboratory was experienced in the first eight years in the analysis, probably due to a series of informative meetings we made over time with the healthcare personnel, aimed at improving their awareness about the importance of a correct laboratory diagnosis in the management of both HAIs and community infections and of the need for close cooperation between clinics and the laboratory to ensure correct patient management. After this period, we observed a remarkable but expected decrease in the absolute number of the samples sent in the two-year pandemic period of 2020–2021, which, however, persisted even in the last years. We can explain the decrease during the pandemic, as the majority of the facility was converted into a structure for the management of low-complexity COVID-19-positive patients that needed respiratory rehabilitation. For this reason, many hospital admissions and planned surgeries were not performed, reducing the number of samples. Nevertheless, an explanation for the findings in the post-pandemic period was not simple to find. We hypothesised that in the number of diagnostic tests performed per patient decreased, aimed at prevention based on diagnostic appropriateness in order to avoid waste of healthcare resources [24]. This result suggests how essential it is to create a trusting relationship among all the professionals involved in healthcare. In light of this gap, we intend to repeat the “ad hoc” meetings on the topic of HAIs and infection control, which were held previously.

In any case, our results highlight an irregular but constant presence of *Staphylococcus* spp. in tested samples, with positivity averaging 10%. This result confirms the common and constant circulation of these microorganisms in healthcare facilities, except for the two years immediately following the COVID-19 pandemic. We can explain this last result by the admitting of a different range of patients susceptible to staphylococcal infections (patients undergoing surgeries, patients affected by vascular/diabetes ulcers, etc.) given the different use of the facility in those years. As expected, considering the medical specialty of the chosen setting, *S. aureus* was by far the most commonly isolated species. We observed that the majority of the affected patients were men in their sixties, often suffering from comorbidities such as diabetes and cardiovascular diseases, in line with scientific evidence that correlate these comorbidities with a higher risk of this kind of infection [25,26]. Many samples came from patients affected by community suppurative infections, such as bone and joint infections, but approximately 20% were samples from patients with an SSI.

Regarding microbial aetiology, it has to be remarked that, despite *S. aureus* being the most commonly detected species, we observed an increased detection of CoNS over time. This result was especially observed for SSIs. Indeed, even though no age and gender differences were found between community infections and SSIs, a very interesting finding was the significant difference detected in the aetiology: CoNS was most commonly detected in SSIs. Although this result could be a consequence of the accidental contamination of the sample, given that these microorganisms are normally present on human skin, this finding is in line with some previous studies in which CoNS accounted for a high percentage of SSIs [27,28,29], confirming the importance of these pathogens as a cause of human infections in some particular conditions. Recent papers highlight the importance of these pathogens in community and nosocomial infections, given the antibiotic resistance often detected in such isolates [30,31,32].

Concerning antibiotic resistance, we observed a general decreasing trend in the detection of antibiotic-resistant strains. However, an increase was observed soon after the pandemic, although we must underline the fact that in the post-pandemic period, the number of samples testing positive for *Staphylococcus* spp. was lower than that in the pre-pandemic period, and this could represent a bias. However, it is well-known that an increase in antibiotic resistance has been observed globally [33], and, therefore, we could hypothesis the possibility of this bias in our study context. This general decreasing trend is however in contrast with global scientific evidence showing an overall increase in the phenomenon of antibiotic resistance and estimates of future deaths expected as a result of this issue [34]. We can assume that this finding could be biased due to the decrease in samples sent to the laboratory that occurred in the last years.

Regarding resistance to specific antibiotic families, first of all it should be emphasised that only a very small number of strains were sensitive to all the antibiotics tested. This suggests that these microorganisms are able to adapt quickly to the presence of antibiotics and highlights the importance of continuous microbiological monitoring in order to gain a clear understanding of the situation regarding antibiotic resistance. Furthermore, it should be emphasised that the vast majority of the isolated strains were found to be MDR. Specifically, we found very high resistance levels to penicillins, quinolones, macrolides, and cephalosporins, in line with global scientific evidence [35]. Specifically, a higher level of resistance was found to the oldest drugs that have been used for a long time. In general, we found very high rates of methicillin resistance in both *S. aureus* (MRSA) and CoNS (MRCoNS), well-known causes of HAIs and community infections [36,37]. Fortunately, low resistance rates to the antibiotics used in the first line against these pathogens (i.e., linezolid, glycopeptides, and tigecycline) were detected. However, a very interesting finding was CoNS being more resistant to these antibiotics (with the exception of tigecycline) than *S. aureus*. This finding confirms the potential role played by CoNS in causing severe and difficult-to-treat infections, as shown in the general literature [38,39]. These linezolid-resistant CoNS (LRCoNS) are able to cause outbreaks. Many studies have highlighted the presence of clonal LRCoNS, such as ST5, ST22, typed through multi-locus sequence typing (MLST), across one or more patients and facilities [40,41,42]. However, regarding tigecycline, our results contrast those already existing, showing very low resistance in both *S. aureus* and CoNS, with only 1.6% the latter showing resistance to this antibiotic [43,44,45].

Our findings highlight the importance of the continuous monitoring of antibiotic resistance rates of staphylococci due to the importance of these pathogens in human pathology and their ability to develop resistance. Especially in hospital settings, this monitoring action is crucial in order to manage the HAIs caused by these common pathogens. It appears essential to prevent these infections through the implementation of the well-known and recommended best practices such as hand hygiene [46,47] and sanitation of operating theatres and invasive items such as endoscopes [48,49] and of the general hospital environment [50].

## 5. Conclusions

*Staphylococcus* spp. are the main bacterial species causing human infections, with a constant presence in community and hospital settings. The development of antibiotic resistance contributes to the already known pathogenicity of some members of this genus. The circulation, especially in hospitals, of certain strains resistant to the antibiotics used to treat MRSA and MRCoNS requires careful and continuous clinical and environmental monitoring in order to detect them and limit their diffusion and the onset of outbreaks. This activity appears essential especially in settings where the risk for this kind of infections is high, such as during orthopaedic and traumatological surgical procedures. New perspectives are necessary to manage this issue. Improving diagnostics, strengthening multidisciplinary collaboration, and increasing research will be the cornerstones for addressing this critical issue in the future.

## Figures and Tables

**Table 1:** Total numbers and percentages of *Staphylococcus* spp.-positive samples relative to the overall number of samples submitted to the laboratory each year.

Years	Total Sent Samples	*Staphylococcus* spp.-Positive Samples (%)
2012	107	14 (13.1%)
2013	143	15 (10.5%)
2014	146	13 (8.9%)
2015	149	9 (6.0%)
2016	201	23 (11.4%)
2017	182	19 (10.4%)
2018	215	21 (9.8%)
2019	242	36 (14.9%)
2020	161	17 (10.6%)
2021	105	11 (10.5%)
2022	104	2 (1.9%)
2023	98	7 (7.1%)
2024	89	9 (10.1%)
TOT	1942	196 (10.1%)

**Table 2:** Percentage resistance to the different generations of penicillins, cephalosporins, and quinolones tested in our study.

PENICILLINS (66.4%)	Mean Percentage of Resistance
Benzylpenicillin (88.3%)	88.3%
Aminopenicillins: - *Ampicillin (84.1%)* - *Amoxicillin (83.0%)* - *Amoxicillin-Clavulanic acid (56.4%)*	74.5%
Penicillinase-resistant: - *Methicillin (78.8%)* - *Oxacillin (70.9%)*	74.8%
Carboxypenicillins: - *Carbenicillin (52.0%)*	52.0%
Ureidopenicillins: - *Piperacillin (45.6%)* - *Mezlocillin (38.2%)*	41.9%
**QUINOLONES (55.7%)**	
I gen. quinolones: - *Cinoxacin (99.0%)*	99.0%
II gen. quinolones: - *Ciprofloxacin (46.4%)* - *Ofloxacin (44.4%)* - *Norfloxacin (50.0%)*	46.9%
III gen. quinolones: - *Levofloxacin (38.8%)*	38.8%
**CEPHALOSPORINS (51.0%)**	
I gen. cephalosporins: - *Cephalexin (65.7%)* - *Cefazolin (52.6%)* - *Cefalotin (45.7%)*	54.7%
II gen. cephalosporins: - *Cefuroxime (43.9%)*	43.9%
III gen. cephalosporins: - *Cefotaxime (56.4%)* - *Ceftriaxone (55.6%)*	56.0%

**Table 3:** Percentage difference between antibiotic-resistant strains detected in SSIs and community infection cases (SSIs = surgical site infections; Δ% = percentage variation in antibiotic resistance between SSIs and community infection; ns = not significant).

	SSIs	Community Infections	Δ%	*p*-Value
Aminoglycosides	30.8	23.1	33.3	ns
Carbapenems	36.7	12.9	184.5	<0.0001
Cephalosporins	61.3	44.9	36.6	0.0333
Chloramphenicol	23.1	22.9	0.9	ns
Co-trimoxazole	8.3	3.5	137.1	ns
Fosfomycin	60.5	54.1	11.8	ns
Glycopeptides	7.8	3.0	160.0	ns
Lincosamides	34.9	30.3	15.2	ns
Linezolid	7.7	2.0	285.0	ns
Macrolides	44.2	54.2	−18.5	ns
Mupirocin	20.0	21.7	−7.8	ns
Nitrofurantoin	7.7	8.3	−7.2	ns
Penicillins	72.3	62.1	16.5	ns
Quinolones	49.6	55.1	−10.1	ns
Rifampicin	20.5	15.3	34.0	ns
Tetracyclines	12.8	7.8	63.8	ns
Tigecycline	15.4	8.3	85.5	ns
Average value	31.6	26.6	/	/

## Data Availability

The raw data supporting the conclusions of this article will be made available by the authors on request.

## Supplementary Materials

The following supporting information can be downloaded at: https://www.mdpi.com/article/10.3390/pathogens14121289/s1, Table S1: List of antibiotics used to determine the antibiotic resistance patterns of isolated staphylococci.
