# Supplementary material for: Staphylococci: What Has Changed in the Antibiotic Resistance Profile in the Last Decade—Analysis of Strains Isolated from Hospitalised Patients
Source: Pathogens. 2025 Dec 15;14(12):1289. doi: 10.3390/pathogens14121289 (PMC12735516; doi:10.3390/pathogens14121289)
Supplement: Supplementary file 1 [file pathogens-14-01289-s001.zip › pathogens-4008089-supplementary.pdf]

CELL WALL INHIBITING AND DISRUPTING MEMBRANE ANTIBIOTICS	**B-LACTAMS**	**PENICILLINS**	*Natural penicillins*	*penicillin (P, 1 unit,)*
			Aminopenicillins	*amoxicillin (AML, 10 µg)*
				ampicillin (AMP, 10 µg)
			Carboxipenicillins	*carbenicillin (CAR, 100 µg)*
			Ureidopenicillins	*mezlocillin (MEZ, 75 µg)*
				piperacillin (PRL, 100 µg)
			Penicillinase-resistant penicillins	*oxacillin (OX, 1 µg)*
				meticillin (MET, 5µg)
			Combinated penicillins	*amoxicillin + clavulanic acid (AMC, 30 µg)*
		**CEPHALOSPORINS**	*1st generation*	*cefazolin (KZ, 30 µg)*
			2nd generation	*cefoxitin (FOX, 30 µg)*
				cefuroxime (CXM, 30 µg)
			3rd generation	*cefotaxime (CTX, 30 µg)*
				ceftazidime (CAZ, 30 µg)
				ceftriaxone (CRO, 30 µg)
		**CARBAPENEMS**	*imipenem* (IMI, 10 µg)	
	**GLICOPEPTIDES**	*vancomycin (VAN, 30 µg)*		
		teicoplanin (TEC), 30 µg)		
	**FOSFOMYCIN**	*fosfomycin ((FO,…)*		
NUCLEIC ACIDS INHIBITING ANTIBIOTICS	**INHIBITING DNA TOPOISOMERASIS ANTIBIOTICS**		**QUINOLONES**	*cinoxacin (CIN, 100 µg)*
			**FLUORQUINOLONES**	*ciprofloxacin (CIP, 5 µg)*
				levofloxacin (LEV, 5 µg)
				norfloxacin (NOR, 10 µg)
				ofloxacin (OFX, 5 µg)
	**INHIBITING FOLIC ACID SYNTHESIS ANTIBIOTICS**		**SULFONAMIDES**	*sulphamethoxazole + trimethoprim* (SXT, 25 µg)
	**INHIBITING RNA SYNTHESIS ANTIBIOTICS**		**RIFAMYCINS**	*rifampicin* (RD, 30 µg)
	**DNA INHIBITORS ANTIBIOTICS**		**NITROFURANS**	*nitrofurantoin* (F, 300 µg)
PROTEIN SYNTHESIS INHIBITING ANTIBIOTICS	**30S SUBUNIT INHIBITORS**		**AMINOGLYCOSIDES**	*gentamycin* (CN, 10 µg)
				sisomicin (SIS, 30 µg)
			**TETRACYCLINES**	*doxycyclin* (DO, 30 µg)
				tetracycline (TE, 30 µg)
				minocycline (MN, 30µg)
			**GLYCYLCYCLINES**	*tigecycline (TGC, 15 µg)*
	**50S SUBUNIT INHIBITORS**		**MACROLIDES**	*azithromycin* (AZM, 15 µg)
				eritromycin (E, 15 µg)
			**LINCOSAMIDES**	*lincomycin* (MY, 2 µg)
				clindamycin (DA,10 µg)
			**OXAZOLIDINONES**	*linezolid* (LNZ, 10 µg)
			**PHENOLIC DERIVATIVES**	*chloramphenicol* (C, 30 µg)

Table S1: List of antibiotics used to determine the antibiotic resistance patterns of isolated staphylococci.
